# 528 Burned While Buzzed: The Impact of Alcohol Intoxication on Stem Cell Cytokines with Major Burns

**DOI:** 10.1093/jbcr/iraf019.157

**Published:** 2025-04-01

**Authors:** Athena Hoppe, Dhanushka Vitharana, William Melito, Paige Deville, Sophia Trinh, Jeffrey Carter, Herbert Phelan, Jonathan Schoen, Victoria Miles, Alison Smith

**Affiliations:** Louisiana State University; Louisiana State University Health Sciences Center - New Orleans; Louisiana State University; Louisiana State University Health Sciences Center - New Orleans; Louisiana State University Health Sciences Center - New Orleans; University Medical Center New Orleans; University Medical Center, New Orleans Burn Unit; Louisiana State University Health Sciences Center - New Orleans; University Medical Center New Orleans; Louisiana State University Health Sciences Center - New Orleans

## Abstract

**Introduction:**

The combined effects of acute ethanol intoxication on the outcomes of patients with severe burn injuries have been documented in earlier studies. However, the underlying molecular mechanisms driving this relationship remain unclear. Adipose-derived stem cells (ADSCs) play a crucial role in promoting the healing of burn wounds by releasing paracrine factors that enhance the wound healing process, including various cytokines. In this research, we aimed to investigate the hypothesis that acute ethanol intoxication at the moment of a burn injury influences the profile of paracrine factors secreted by ADSCs derived from affected adipose tissue.

**Methods:**

Adipose tissue was collected from adult patients with severe burn injuries at the index operation (n=28). ADSCs were extracted and cultured in vitro. Supernatants were harvested 30 hours after plating and used for cytokine determinations by Multiplex assay. Fluorescence activated single cell sorting (FACS) confirmed their phenotype with markers CD 90, CD 166, and CD 73. Univariate analyses were performed to compare two cohorts, patient with ethanol intoxication (n=15) vs those with no ethanol intoxication (n=15) at time of burn.

**Results:**

Blood ethanol concentrations averaged 121 +/- 41.7 mg/dL. The analyte assay demonstrated a significant decrease noted of fibroblast growth factor (FGF)-2 in patients with elevated blood ethanol content. No significant differences in supernatant concentrations of IL-1 beta, IL-6, IL-8, and IL-13 were observed (p>0.05)

**Conclusions:**

The results from this study suggest that ethanol intoxication at the time of major burn injury may alter the profile of paracrine factors secreted from ADSCs, especially as this relates to FGF-2 which has been shown to contribute to many cell functions including smooth muscle cell growth, wound healing, and cell migration. These results highlight the need for additional analysis of the effects of alcohol intoxication on long term healing potential of burn wounds.

**Applicability of Research to Practice:**

Understanding the importance of cytokine changes in patients with elevated blood alcohol level may allow clinicians to direct resuscitation and treatment with additional intentionality

**Funding for the Study:**

N/A

